# Soybean peroxidase-mediated degradation of an azo dye– a detailed mechanistic study

**DOI:** 10.1186/1471-2091-14-35

**Published:** 2013-12-05

**Authors:** Liaquat Ali, Rowdha Algaithi, Hosam M Habib, Usama Souka, Muhammad A Rauf, S Salman Ashraf

**Affiliations:** 1 Department of Nutrition and Health, College of Food and Agriculture, UAE University, P.O. Box 15551, Al-Ain, United Arab Emirates; 2 Department of Chemistry, College of Science, UAE University, P.O. Box 15551, Al-Ain, United Arab Emirates

**Keywords:** Azo dye, Degradation, Enzyme, Mediator, LC/MS, Metabolites

## Abstract

**Background:**

Peroxidases are emerging as an important class of enzymes that can be used for the efficient degradation of organic pollutants. However, detailed studies identifying the various intermediates produced and the mechanisms involved in the enzyme-mediated pollutant degradation are not widely published.

**Results:**

In the present study, the enzymatic degradation of an azo dye (Crystal Ponceau 6R, CP6R) was studied using commercially available soybean peroxidase (SBP) enzyme. Several operational parameters affecting the enzymatic degradation of dye were evaluated and optimized, such as initial dye concentration, H_2_O_2_ dosage, mediator amount and pH of the solution. Under optimized conditions, 40 ppm dye solution could be completely degraded in under one minute by SBP in the presence of H_2_O_2_ and a redox mediator. Dye degradation was also confirmed using HPLC and TOC analyses, which showed that most of the dye was being mineralized to CO_2_ in the process.

**Conclusions:**

Detailed analysis of metabolites, based on LC/MS results, showed that the enzyme-based degradation of the CP6R dye proceeded in two different reaction pathways- via symmetric azo bond cleavage as well as asymmetric azo bond breakage in the dye molecule. In addition, various critical transformative and oxidative steps such as deamination, desulfonation, keto-oxidation are explained on an electronic level. Furthermore, LC/MS/MS analyses confirmed that the end products in both pathways were small chain aliphatic carboxylic acids.

## Background

Extensive use of synthetic dyes and their subsequent release in industrial wastewater is a growing environmental problem [1]. It is estimated that as much as 12% of the dyestuff amounting to about 280,000 ton**/**year is released to the ecosystem [2]. These dyes are used in various industrial applications (total consumption is more than one million tons of dyes annually) and are engineered to be generally resistant to fading. They not only need to sustain alkaline or acidic environment but also need to withstand washing with soaps and bleaching agents and be resistant to light and ultraviolet irradiation.

It is well-established that some dyes are potentially carcinogenic and mutagenic, as well as genotoxic [3-5]. Furthermore, the presence of color in water bodies causes less sunlight to penetrate through water which results in reducing the photosynthetic capability of aquatic plants and microorganisms [6]. Industrial effluents containing dyes are able to color water even in concentrations as low as 1 mg/liter, whereas in most cases, these wastewater streams typically contain a much higher amount of the dye content, about 10-200 mg/liter, which gives intense coloration. It is therefore not surprising that both international and national regulations for industrial wastewater require substantial elimination of the dyestuff content form the effluent [7,8]. Numerous approaches have been developed to treat industrially generated wastewater, such as coagulation/flocculation, adsorption, use of activated carbon, and various forms of Advanced Oxidation Processes, e.g. ozonation, and photochemical approaches [9-16]. Most of these methods have a disadvantage of either formation of large amounts of sludge or production of possible toxic byproducts.

Removal of contaminants from the environment by biological methods using enzymes and microorganisms is considered to be closer to nature as it is an environmentally friendlier technique which leaves the ecosystem intact [17]. The technology is scalable and can also be used to treat other organic impurities [18]. A number of microorganisms including bacteria, fungi, and yeasts have been used to treat the dye contaminated wastewaters [19-22].

Azo dyes are electron-deficient xenobiotic components because of their azo linkage (–N=N), and in many cases, they have sulphonic (SO_3_^−^) or other electron-withdrawing groups, which generate an electron deficiency and make the dye less susceptible to degradation by microorganisms. However, under appropriate conditions, they can be degraded by azo reductase-releasing microbes.

Enzyme mediated decolorization is another newer alternative, where the enzyme can specifically react with organic pollutants by transforming them into low molecular weight products [23]. The main advantage of using enzymes in degrading dye solutions is that they have higher reaction rates and can also work in milder reaction conditions. Some azo dyes have been decolorized by using certain peroxidases, such as soybean peroxidase (SBP), manganese peroxidase (MnP), lignin peroxidase (LiP), laccase and horseradish peroxidase (HRP) [23-26]. It has been suggested that these enzymatic treatments could oxidize the dye structures to form compounds with lower molecular weight and lower toxicity and may eventually mineralize the dyes.

Although a considerable amount of research has been published on the use of pure enzymes to degrade dyes, detailed analyses of the breakdown pathway are almost non-existent. Structures of the intermediates produced as well as the mechanisms involved in the dye degradation pathway are important to properly understand and further exploit this novel approach to pollutant-contaminated water remediation.

The objective of the present study was to study in detail the mechanism of an azo-dye degradation by soybean peroxidase enzyme (SBP). In addition to identifying the various intermediates produced and proposing possible pathways, factors affecting dye degradation such as initial dye concentration, H_2_O_2_ concentration, pH, and presence of redox mediator were also evaluated. The present study is one of the very few studies that show in detail the various intermediates produced during peroxidase-mediated degradation of an azo-dye and possible electron-level mechanisms involved.

## Methods

### Reagents

The azo dye namely Crystal Ponceau 6R (C.I name = Acid Red 44, C.I number = 16250, Molecular Formula = C_20_H_12_N_2_O_7_S_2_Na_2**,**_ FW = 502.446 g mol^−1^), herein abbreviated as CP6R was used as a model dye. The dye was procured from Sigma-Aldrich Chemicals and used as such. All the other chemicals used in this work were obtained from Sigma-Aldrich and were of high purity (< 98%).

### Dye degradation studies

Stock solution (2,000 ppm) of the dye was prepared in a 250 mL flask by first dissolving an appropriate amount in deionized water. Further dilutions from this stock were done as per the requirement of the experiment. Unless otherwise indicated, the working concentration of the CP6R dye was 40 ppm. Dye degradation reactions were carried out by adding H_2_O_2_ to a buffered dye solution containing SBP enzyme. Spectrophotometric measurements were made using a CARY 50 UV/Vis spectrophotometer. The absorbance value obtained in each case was plotted against time to obtain the % degradation. The % degradation for the dye was calculated by observing the changes in λ_max_ (510 nm) of the solution. The studies were carried out at 25°C otherwise indicated. For pH studies, the dye solution were prepared in 33.33 mM universal buffers (citrate-phosphate) adjusted to specific pH value.

The absorption spectrum of an aqueous solution of Crystal Ponceau 6R (CP6R) was scanned in the range of 200-800 nm with a λ_max_ value appearing at 510 nm. The change in intensity of the λ_max_ value was used to calculate the percentage dye remaining as well as the percentage change of dye degradation as follows:

(1) $$\%\;\mathrm{dye}\;\text{remaining}=\left(\mathrm{At}/\mathrm{Ao}\right)\;\times \;100$$

(2) $$\%\;\text{degradation}=\left[\left(\mathrm{Ao}-\mathrm{At}\right)/\left(\mathrm{Ao}\right)\right]\;\times \;100$$

Where *A*_*0*_ is the initial absorbance of dye solution and *A*_*t*_ is the absorbance of the dye solution at any given time.

### Total Organic Carbon (TOC) analyses

TOC analyses were carried out using GE Sievers InnoVox TOC analyzer properly calibrated with fresh standards. The CP6R samples tested were 0% degradation sample which consisted of 400 ppm CP6R in 33 mM citrate-phosphate buffer, pH 4, 0.78 μM SBP, 0.1 mM HOBT and 100% degradation sample which was exactly the same as the 0% sample but contained 1 mM H_2_O_2_. Analyses were carried out in triplicates and the data is presented as TOC values normalized to 0% CP6R degradation sample.

### HPLC and LC/MS experiments

High performance liquid chromatography (HPLC) and LC/MS analyses were carried out similar to as previously described [14]. Briefly, samples were analyzed on an Acquity UPLC system, (Waters Corporation, Milford, MA, USA) with an Acquity UPLC BEH C_18_ column with 1.7 μm particle size (2.1 mm I.D. × 100 mm length, Waters Corporation, Milford, MA, USA) maintained at 35°C, coupled to Acquity tunable ultraviolet/visible detector (Waters Corporation, Milford, MA, USA) and an Acquity Tandem quadruple mass spectrometer (Waters Corporation, Milford, MA, USA). The mobile phase consisted of solution A (0.1 M ammonium formate (pH 6.7)) and solution B (1:1 acetonitrile/methanol) and a gradient of 0% B to 80% B in 13.80 minutes at the flow rate was 0.2 mL/min was used to obtain the chromatographs. The mass spectrometer was equipped with an electrospray ionization source operated in negative ion mode. The ESI conditions were as follows: capillary voltage: 3.0 kV, Cone voltage 30 V, collision energy 50 V, desolvation gas (Nitrogen at 500 L/Hr), Cone gas (Nitrogen at 2 L/Hr), desolvation temperature was set at 350°C and source temperature was 150°C. The mass range was detected from 50 to 700 m/z. Tandem MS experiment was done using Waters Masslynx V 4.1, wherein Argon gas was used as the collision gas.

## Results and discussion

### Optimizing the enzymatic dye degradation reaction conditions

As expected, the degradation of dye was found to be very much affected by the initial amount of dye content in solution. Studies carried out at different concentrations of the dye showed the optimum CP6R under our chosen conditions was 40 ppm (data not shown). Optimizations of other parameters are described below:

### Requirement of HOBT for SBP-mediated degradation of CP6R

Initial experiments using only SBP and H_2_O_2_ showed that unlike other dyes, CP6R was unable to be degraded by SBP/H_2_O_2_ alone (data not shown). It is well known that the presence of redox mediators such as 1-hydroxybenzotriazole (HOBT), veratryl alcohol, violuric acid, 2- methoxyphenothiazone, etc. can dramatically increase the rate of dye degradation [27-30]. The mechanism involved is well known, wherein, the substrate initially undergoes an one-electron oxidation in the presence of a redox mediator and transforms into a radical cation followed by abstraction of a H-atom from the substrate by the mediator and converting it into a radical, which can then cause the substrate to co-oxidize [30].

**Figure 1:**
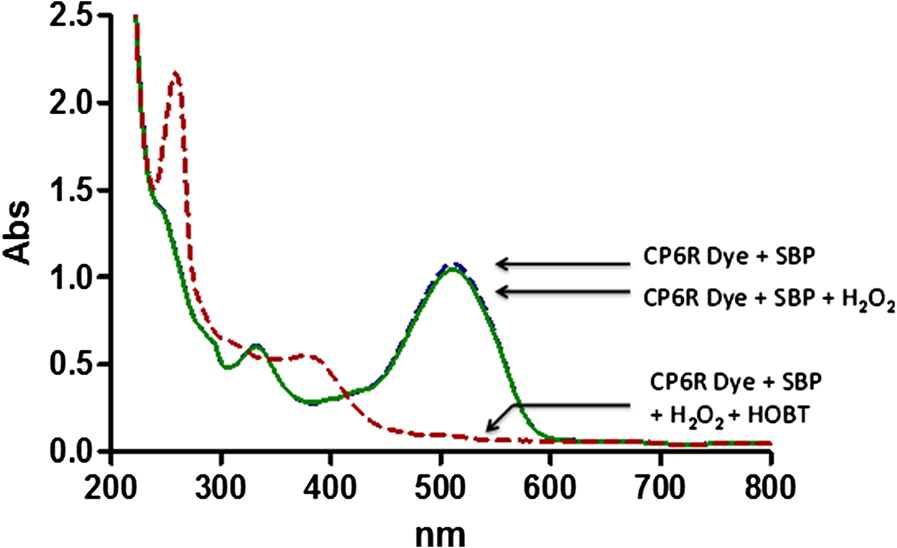
**Absolute requirement of a redox mediator (HOBT) for SBP-H**_**2**_**O**_**2**_**mediated CP6R degradation.** Full UV-Vis scans of 1) CP6R (30 ppm in pH 5 buffer) with 0.32 μM SBP, 2) CP6R with 0.32 μM SBP and 0.1 mM H_2_O_2_, and 3) CP6R, 0.32 μM SBP, 100 μM H_2_O_2_ and with 0.1 mM HOBT (after 1 minute) are shown.

Since CP6R could not be degraded by SBP enzyme and H_2_O_2_ alone, we decided to include HOBT in the reaction. As can be seen in Figure 1, the presence of HOBT resulted in the dramatic and immediate degradation of the CP6R. In order to determine the optimum concentration of HOBT required for efficient CP6R degradation by SBP/H_2_O_2_, a systematic study was carried out by using increasing concentrations of the mediator. As can be seen from Additional file 1: Figure S1, the dye showed significant degradation even at the low concentration of HOBT (25 μM), whereas, at higher HOBT concentrations, the dye underwent very rapid degradation. Even though 100 μM showed almost complete dye degradation by 3 minutes, we chose 50 μM HOBT for further experiments, so that we could see the positive effects of optimizing other parameters.

### Effect of hydrogen peroxide concentration on dye degradation

Since the peroxidase enzymes use H_2_O_2_ as a co-reactant, if the concentration of hydrogen peroxide used is too low, the enzyme activity becomes low; however, a very high peroxide concentration can irreversibly oxidize the enzyme and cause its inactivation [26]. In this regard, experiments were carried out to optimize the H_2_O_2_ concentration while keeping the other parameters constant. The results obtained are shown in Additional file 2: Figure S2, which showed the optimum H_2_O_2_ concentration to be 0.175 mM. Additionally, it can also be seen that at very high H_2_O_2_ concentrations, a significant reduction in dye degradation is observed (due to H_2_O_2_-mediated inactivation of SBP).

### Effect of SBP enzyme concentration on dye degradation

Degradation of dye depends on the amount of catalyst added and the contact time. Thus experiments were also done to optimize the concentration of the enzyme in dye solution which was varied in the range of 0 to 2.7 μM while keeping the other parameters constant. The results are shown in Additional file 3: Figure S3 and it can be noted that the enzyme dose had a significant effect on dye decoloration. At lower SBP concentrations, the dye degradation was not very significant, whereas at very high SBP concentration, the dye degraded very quickly in a very short time (almost 30% in a few seconds). Based on these data, an optimized concentration of 0.27 μM SBP was chosen for all subsequent reactions.

### Effect of pH

Enzymatic driven reactions are known to be pH dependent [28,29]. Thus experiments were done to optimize this parameter as well. SBP mediated dye degradation was studied at different pH values (from 2 to 9), while keeping the other conditions constant. The results are shown in Additional file 4: Figure S4, which shows the dramatic effect of pH on SBP-mediated degradation of CP6R, with the enzyme being most active in the pH 3-5 range. A pH value of 5 was used for all the subsequent experiments. This role of pH on the peroxidase driven reactions has been reported in the literature for different dye degradation along-with its mechanism [17,23].

### Total Organic Carbon analysis

**Figure 2:**
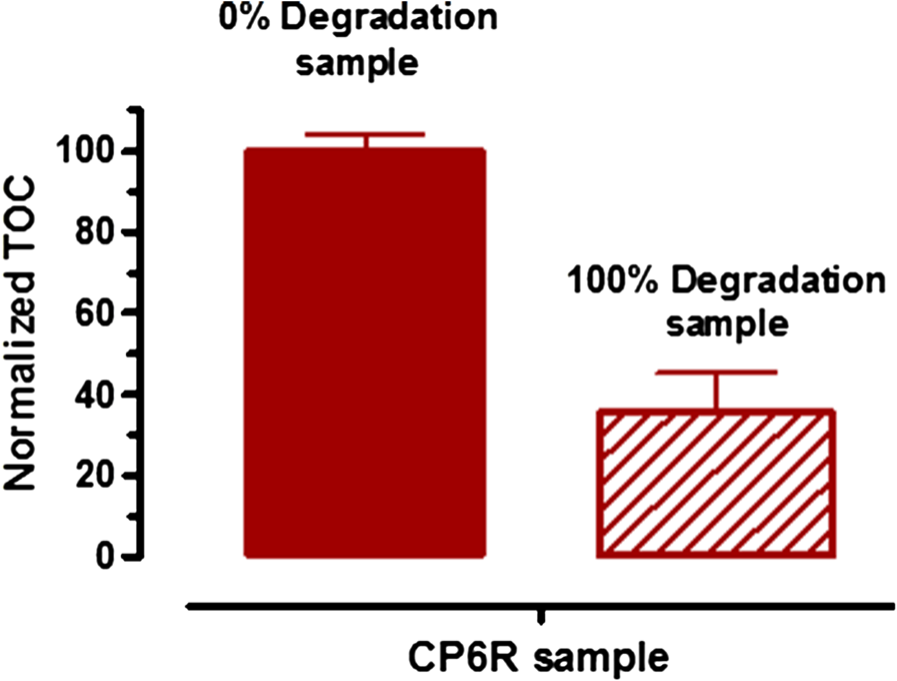
Normalized Total Organic Carbon (TOC) in “0% CP6R degradation” and “100% CP6R degradation” samples, showing the decrease in TOC upon dye decoloration.

In order to confirm that in fact dye degradation was going on and that some of the CP6R was being mineralized to CO_2_, we carried out Total Organic Carbon (TOC) analyses on the pure CP6R dye (in the presence of buffer, HOBT and SBP) as well as a CP6R sample that was 100% degraded based on UV-Vis measurements. As can be seen from Figure 2, there was a significant decrease in TOC (64.5% decrease) in the100% degraded sample, thus confirming mineralization of most of the CP6R during the SBP-mediated remediation of this azo dye. Similar decrease in TOC values upon peroxidase treatment have been reported by other groups as well [27].

### Analysis of product formation using HPLC/MS

**Figure 3:**
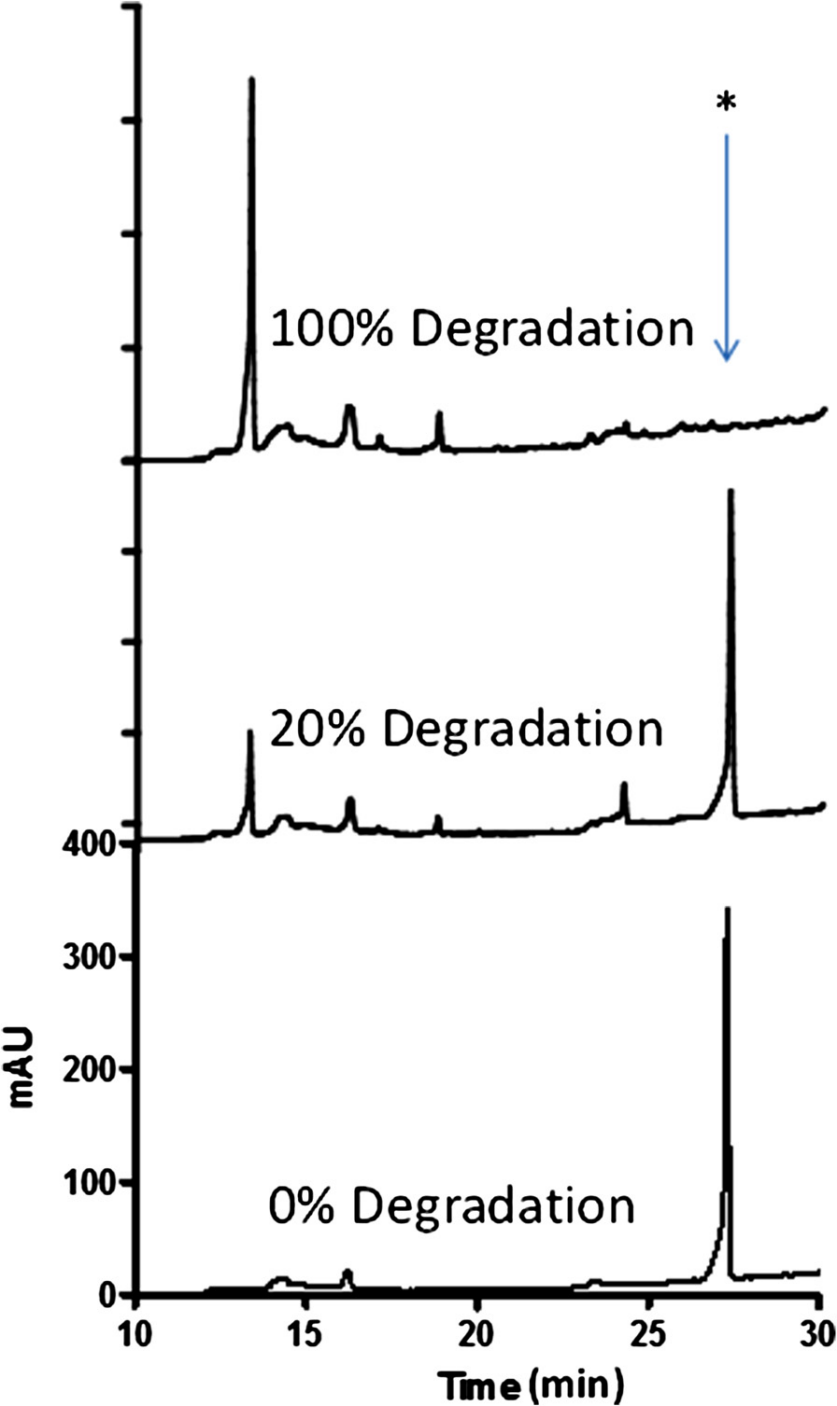
**Reverse Phase HPLC chromatograms of SBP-mediated degradation of CP6R.** The sample labeled “0% degradation” contained CP6R dye, HOBT and SBP (but no H_2_O_2_).

Studies related to the degradation pathway and product identification of the enzymatically driven reactions is of special interest to many researchers working in the area of environmental sciences. Literature survey revealed that several studies have been carried out to determine the mechanism for azo dye degradation [31,32]. However, all of these studies lack detailed electronic-level mechanisms involved in these processes. In order to confirm the degradation of the dye and analyze the product formation, SBP-mediated degradation of CP6R was monitored by Reverse Phase HPLC Diode Array Detector (RP-HPLC-DAD) analysis. The chromatograms of the untreated and treated dye samples are shown in Figure 3. As can be seen from the figure, 20% dye degraded sample showed the breakdown of CP6R into new compounds (new peaks). This was more markedly seen with almost 100% dye degradation sample where the initially formed products were further converted into secondary products. The identity of the breakdown products were confirmed using LC/MS/MS, which are discussed later in this paper.

### Proposed mechanism of enzymatic degradation

The basic scheme of peroxidase-mediated degradation of organic compounds is well established as shown below [30,33]:

$$\mathrm{Fe}\left(\mathrm{III}\right)\text{SBP}+H_{2}O_{2}\;\to \;\left[\mathrm{Fe}\left(\mathrm{IV}\right)=O\right]\mathrm{SB}\overset{•}{P}+H_{2}O$$

$$\frac{\left[\mathrm{Fe}\left(\mathrm{IV}\right)=O\right]\mathrm{SB}\overset{•}{P}+\text{HOBT}}{\text{Compound}\;I}\;\to \;\frac{\;\left[\mathrm{Fe}\left(\mathrm{IV}\right)=O\right]\mathrm{SB}\overset{•}{P}+\text{HOBT}}{\text{Compound}\;\mathrm{II}}$$

$$\frac{\left[\mathrm{Fe}\left(\mathrm{IV}\right)=O\right]\mathrm{SBP}+\text{HOBT}}{\text{Compound}\;\mathrm{II}}\;\to \;\mathrm{Fe}\left(\mathrm{III}\right)\text{SBP}+\overset{•}{H}\text{OBT}+H_{2}O$$

$$\overset{•}{H}\text{OBT}+\mathrm{CP}6R\;\to \;\text{HOBT}+\overset{•}{C}P6R$$

The generation of CP6R radical by SBP in the presence of a mediator (HOBT) consists of four major steps. In the first step, SBP enzyme reacts with H_2_O_2_ to become an oxyl-ferric (Fe^4+^) cation radical, compound I (via loss of two electrons). The second step involves the abstraction of hydrogen from HOBT resulting in the formation of HOBT radical and compound II. In the third step, a second radical of HOBT is formed by the transfer of another hydrogen to compound II, leading to the regeneration of the original reduced (Fe^3+^) SBP enzyme and a water molecule. In the final step, HOBT radical attacks CP6R and abstracts a hydrogen, resulting in the formation of CP6R radical. Similar reactions have been previously well-documented [33].

### Proposed mechanistic pathway of CP6R degradation by SBP-H_2_O_2_

Previous studies on the mechanism of azo dyes degradation have shown two main routes for dye degradation, namely the symmetrical azo bond cleavage, and the asymmetrical azo bond cleavage, both of which can take place at the same time [14,34]. In our present work, we used MS/MS experiments to analyse the various intermediates of CP6R degradation by SBP. The mass of all intermediates observed in the present study are listed in Tables 1 and 2. Detailed analyses (discussed below) indicate that both symmetric and asymmetric azo bond cleavage were involved in SBP-mediated CP6R degradation.

**Table 1:** Mass of all intermediate in asymmetrical degradation

**Compound name**	**Mass (m/z)**	**IUPAC name**
CP6R	457	
A1	317.9	7,8-dihydro-7,8-dixonaphthalene-1,3disulfonic acid.
A2	351.9	2-carboxyvinyl -4,6disulfobenzonic acid.
A3	104.0	Malonic acid.
A4	156.0	3-methylhexa 2,4-diene dionic acid.
B1	155.0	2-(naphthalene-1-yl) diazene.
B2	144.0	Naphthalen-1-ol.
B3	162.0	4-phenyibut-3enoic acid.

**Table 2:** Mass of all intermediate in symmetrical degradation

**Compound name**	**Mass (m/z)**	**IUPAC name**
CP6R	457	
C1	318.9	8-amino-7-hydroxynathphalene-1,3-disulfonic acid.
A1	317.9	7,8-dihydro-7,8-dixonaphthalene-1,3disulfonic acid.
A2	351.9	2-carboxyvinyl -4,6disulfobenzonic acid.
A3	104.0	Malonic acid.
A4	156.0	3-methylhexa 2,4-diene dionic acid.
D1	143.0	Naphthalene-1-amine.
B2	144.0	Naphthalen-1-ol.
B3	162.0	4-phenyibut-3enoic acid.

### Asymmetrical azo bond cleavage of CP6R

**Figure 4:**
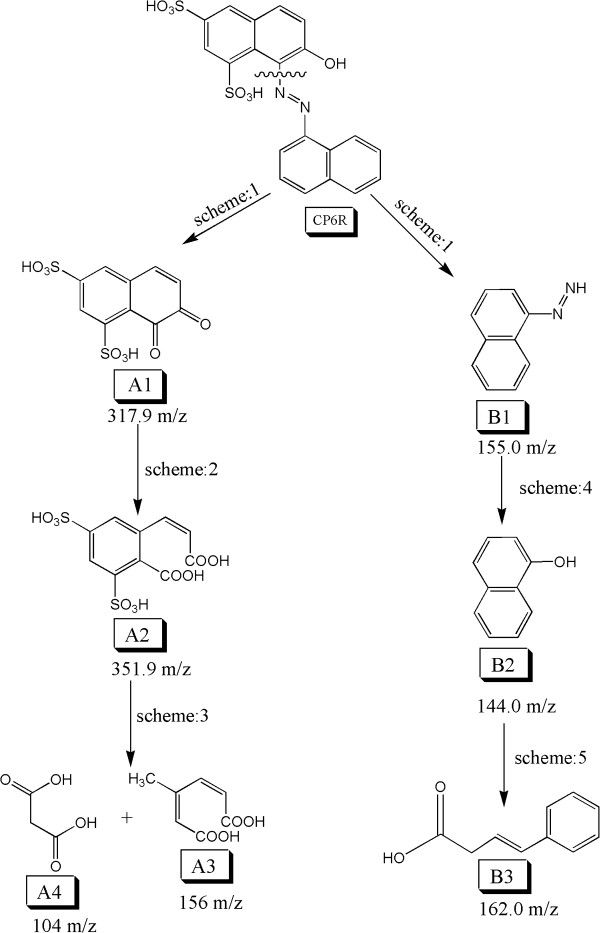
Proposed mechanism for the asymmetrical azo bond cleavage of CP6R by SBP enzyme.

Figure 4 summarizes the asymmetrical azo bond cleavage of CP6R by SBP. Breakage of the azo bond in CP6R may proceed via two mechanistic pathways. The first one involves the C-N bond cleavage on the side that has un-substituted naphthalene. In the present study, no evidence (MS fragment) of this type of asymmetricazo bond cleavage was observed. It seems that SBP enzyme mediates the asymmetrical cleavage of the C-N bond which is closer to the substituted naphthalene. CP6R is initially broken into two main species, namely (A1) (7,8-dihydro-7,8-dixonaphthalene-1,3disulfonic acid) and (B1) (2-(naphthalene-1-yl) diazene). The intermediate (A1), containing sulfonate group will stabilize the benzene ring more as compared to the un-substituted benzene ring. This is due to the electron withdrawing nature of the sulfonate group resulting in the stability of the intermediate as previously reported in the literature [35]. The other side of the intermediate (A1) containing the diketone makes the structure rigid, as a result of which the stability of the intermediate increases. Product (A1) is spontaneously converted to (A2) (2-carboxyvinyl-4,6 disulfobenzonic acid), which is formed by the conversion of keto groups to carboxylic groups. Such enzymatic transformation with ring opening has also been previously reported [36]. Further degradation of (A2) is proposed to produce low molecular weight carboxylic acids, (A3) (3-methylhexa-2,4-dienedioic acid) and (A4) (malonic acid). The azo group of intermediate (B1) can also be radically transformed to an alcohol group to produce (B2) (Naphthalen-1-ol), as has been previously reported in the literature [36]. The intermediate (B1) is stabilized due to resonance in naphthalene, but it is less stable as compared to the other intermediate (B2), because the presence of azo group in B1 will acquire further stability after losing the nitrogen atoms as N_2_ molecule, which results in B2. The species (B2) can then subsequently undergo ring opening to produce a carboxylic acid (B3) (4-phenyibut-3enoic acid).

### Symmetrical azo bond cleavage of CP6R

**Figure 5:**
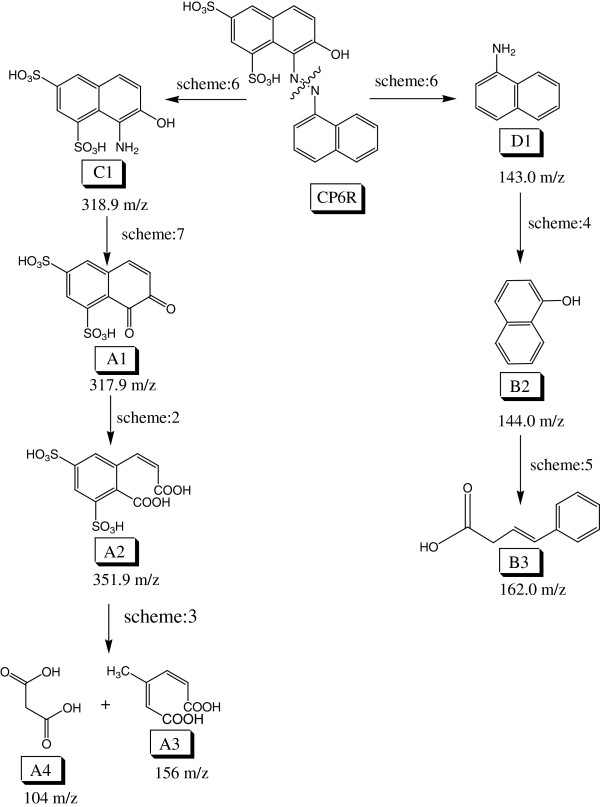
Proposed mechanism for the symmetrical azo bond cleavage of CP6R by SBP enzyme.

Figure 5 summarizes the symmetrical azo bond cleavage of CP6R by SBP. As it can be seen from this scheme, CP6R symmetrical bond cleavage takes place at -N=N- site resulting in two main products namely (C1) (8-amino-7-hydroxynathphalene-1,3-disulfonic acid) and (D1) (2-(naphthalene-1-amine). Intermediates (D1) and (C1) both undergo the same transformation as in asymmetrical cleavage (Figure 4) to produce (B2) (Naphthalen-1-ol) and (A1) (7,8-dihydro-7,8-dixonaphthalene-1,3disulfonic acid), respectively.

### Detailed electronic-level mechanism of asymmetrical cleavage of the azo bond

**Figure 6:**
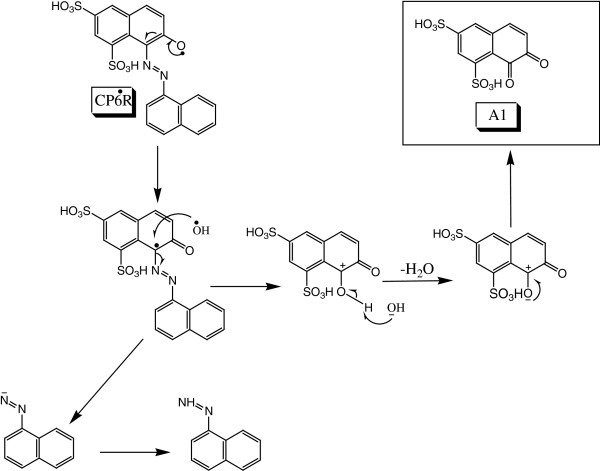
Proposed mechanism for the asymmetrical azo bond cleavage of CP6R by SBP enzyme and subsequent conversion to a diketo intermediate (A1).

Figure 6 shows a proposed detailed electronic-level mechanism for the asymmetric azo bond cleavage, which starts with the formation of CP6R radical by abstraction of a hydrogen. Soybean peroxidase prefers the abstraction of hydrogen from hydroxyl group rather than sulphur group, because oxygen is more electronegative than sulfur [37]. Thereafter, the radical shifts to the carbon which is attached to the nitrogen, resulting in the formation of a ketone. This leads to the breakdown of the π bond and the conjugation of the azo group, thus making it less stable. The OH radical attack on the carbon atom attached to the nitrogen results in the detachment of the azo group and the formation of diketo intermediate (A1) and -N=N-aryl intermediate (B1).

### Oxidation of diketo

The oxidation of diketo to carboxylic acid has been observed in several photolytic degradation studies [38]. The ring is initially opened by the attack of OH radical on alpha diketones resulting in the formation of dicarboxilic acid intermediate (A2). This is shown in the Additional file 5: Figure S5.

### Desulfonation reaction

**Figure 7:**
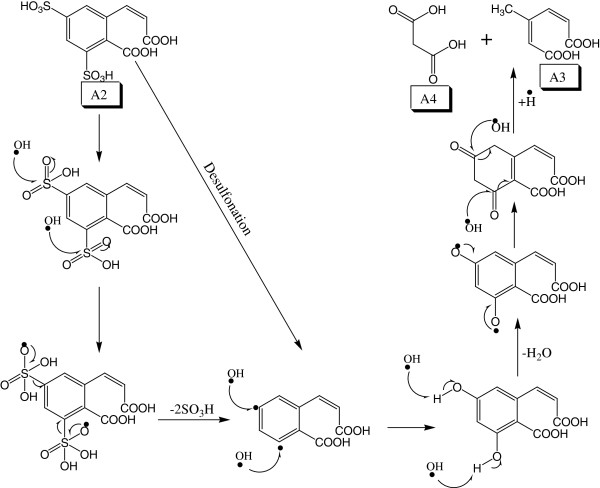
Proposed mechanism for the desulfonation of intermediate A2 to small molecular weight carboxylic acids A3 and A4.

During the degradation of CP6R molecule, desulfonation of the two aryl sulfonic groups occurs readily from the (A2) intermediate of the dye. Due to the extended orbitals of sulfur atom, the OH radical preferentially attacks this atom, resulting in the eventual cleavage of the C-S bond of the sulfonic group [38-40]. Furthermore, the second sulfur atoms in A2 can be attacked by another OH radical simultaneously resulting in the removal of both of the –SO_3_H groups and the formation of radical species. This is followed by subsequent oxidation steps and resulting in low molecular weight carboxylic acid molecules (A3 and A4) (Figure 7).

### Deamination and removal of azo group

**Figure 8:**
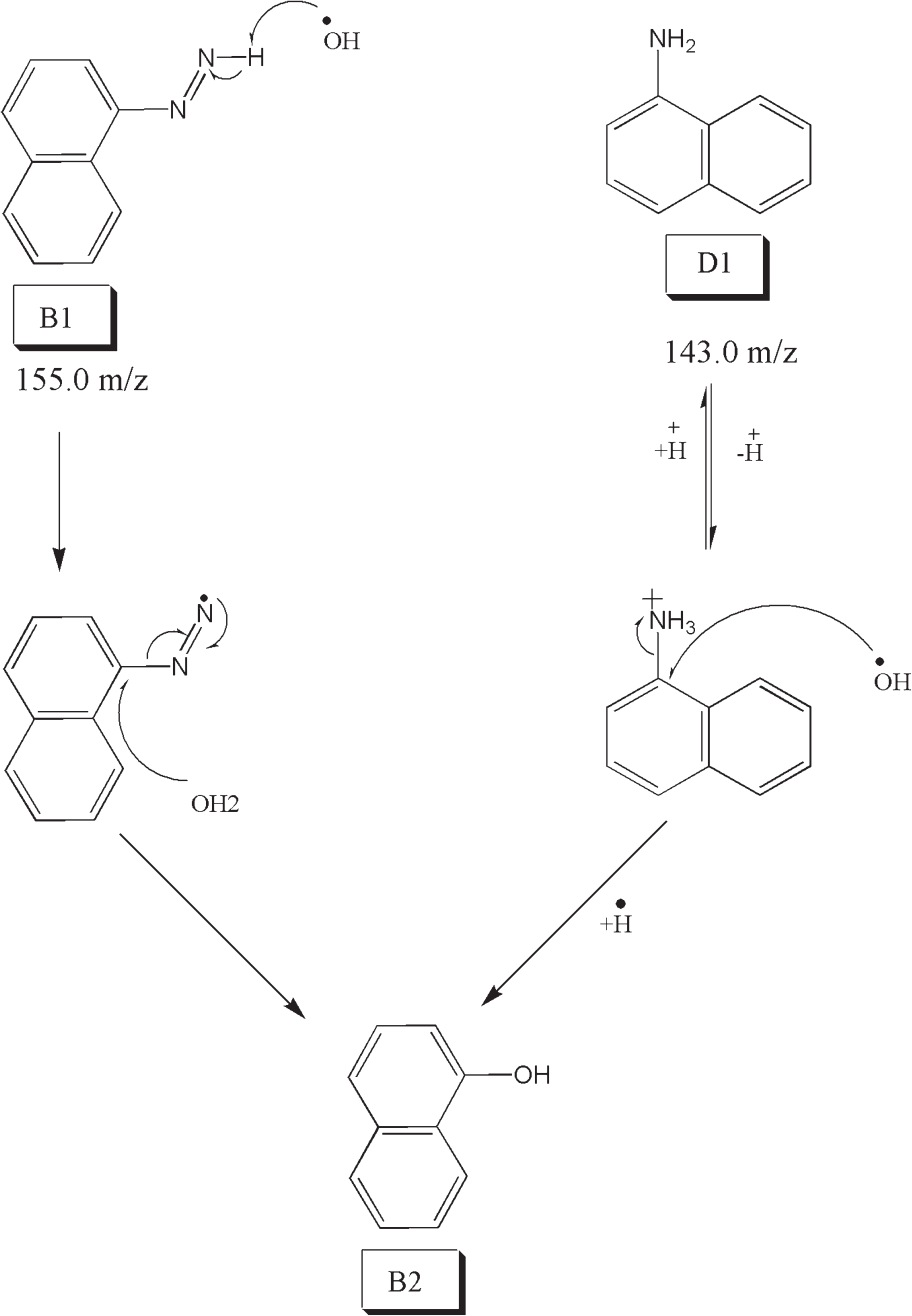
Proposed mechanism for the deamination of intermediate D1 and the subsequent removal of the azo group of intermediate B1 to produce a phenol.

Symmetrical degradation of CP6R produces phenyl diazene intermediate (B1). The OH radical abstracts hydrogen from nitrogen, followed by radical shift to the nitrogen resulting in the formation of phenol by releasing nitrogen molecule. Similar conversion has been observed for Methyl Orange dye [32]. The asymmetrical degradation shows aromatic amine intermediate (D1), which is different than what is produced during symmetrical degradation. The aqueous media of CP6R dye solution provides the protonated hydrogen to form NH_3_^+^, after which the OH radical attacks on carbon containing NH_3_^+^ results in the formation of a phenol [41]. This scheme is shown in Figure 8.

### Oxidation of phenol

**Figure 9:**
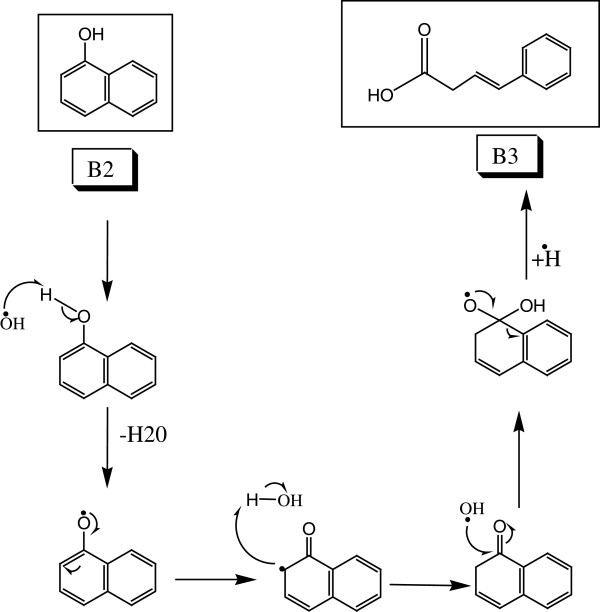
Proposed mechanism for the oxidation of intermediate B2 to a carboxylic acid B3.

As shown in Figure 9, degradation of intermediate phenol (B2) can take place by the abstraction of hydrogen radical from the phenolic group of B2, resulting in the formation of radical on oxygen. This is followed by the formation of ketone which is further oxidized to carboxylic acid (B3).

### Formation of amine in symmetrical cleavage

**Figure 10:**
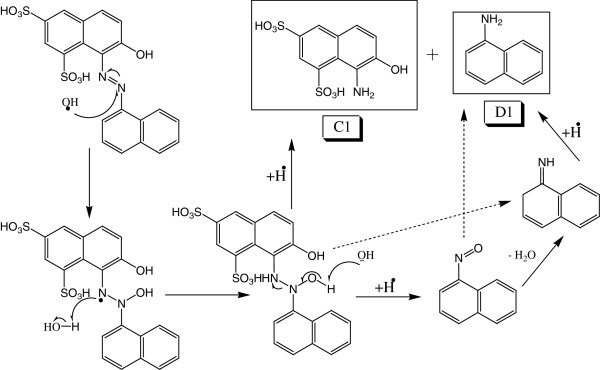
Proposed mechanisms for the symmetrical azo bond cleavage and generation of intermediates C1 and D1.

The OH radical prefers to attach on the nitrogen of azo group because of double bond. Several studies have reported that the cleavage of N=N group can produce amine and ketoamine [14,42]. During symmetrical degradation of CP6R, (C1) and (D1) intermediates are formed as shown in Figure 10. The formation of amine on substituted naphthalene (C1) is due to the attack of hydrogen radical. In the case of intermediate (D1), the formation of amine can occur via a couple of different ways. One of the approaches is the conversion of the unsubstituted napthalene into nitrosonaphthalene which has been previously reported [14], which then is converted into ketoamide, and can then abstract hydrogen (or H radical) and form D1. Alternatively, nitrosonaphthalene may form D1 directly (without going through the ketoamide intermediate) [42]. The other possibility is the conversion of the unsubstituted naphthalene directly to ketoamide, which then is converted into D1.

### De-amination in symmetrical cleavage

**Figure 11:**
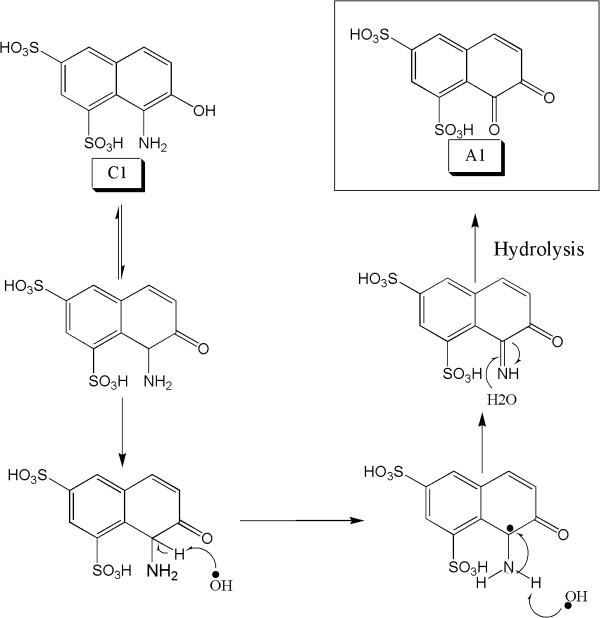
Proposed mechanism for the enzymatic deamination of intermediate C1 to eventually generate the diketo intermediate A1.

Enzymatic degradation of CP6R produces the amine intermediate (C1). The oxidation of un-substituted aromatic amine is difficult as compared to substituted aromatic amine. In CP6R, aromatic amine intermediate is formed containing the phenol group at the ortho position. This aminophenol undergoes a keto-enol equilibrium. As shown in Figure 11, the OH radical then abstracts acidic alpha hydrogen of the tautomer, resulting in the formation of ketoamine, which is further hydrolyzed to diketo. Such de-amination has been reported in photolytic degradation of Amido Black and RB [14].

## Conclusion

In summary, we show here efficient degradation of an azo-dye, Crystal Ponceau 6R (CP6R), using the Soybean peroxidase/HOBT/H_2_O_2_ system. Under optimized conditions it was found that SBP could degrade 100% of the dye in under a minute. Dye mineralization was confirmed using TOC and HPLC experiment, and most importantly, extensive LC/MS/MS experiments were used to identify the various metabolites formed during the degradation process. Lastly, based on the LC/MS data and known radical-based reactions, we were able to develop detailed mechanisms for the various steps in the dye degradation. Our results show that the azo dye degraded via two different pathways, namely symmetric and asymmetricazo bond cleavage followed by diketo oxidation to carboxylic acids, desulfonation, deamination, and phenolic oxidation reactions.

## Competing interests

All authors declare that they have no competing interests in this work.

## Authors’ contributions

LA, HMH, and US carried out the LC/MS/MS analyses. LA elucidated the detailed mechanisms for the peroxidase mediated dye degradation. RA carried out the dye degradation optimization studies. MAR and SSA wrote the manuscript and supervised the work. SSA conceived and planned the whole project. All authors have read and approved the final manuscript.

## Supplementary Material

Additional file 1: Figure SI Effect of HOBT concentration of SBP/H_2_O_2_ mediated degradation of CP6R. [CP6R] = 40 ppm, [SBP] = 032 μM, [H_2_O_2_] = 0.1 mM. Click here for file

Additional file 2: Figure S2 Effect of H_2_O_2_ concentration on SBP/H_2_O_2_/HOBT mediated degradation of CP6R. [CP6R] = 40 ppm, [SBP] = 032 μM, [HOBT] = 50 μM. Click here for file

Additional file 3: Figure S3 Effect of SBP enzyme concentration on SBP/H_2_O_2_/HOBT mediated degradation of CP6R. [CP6R] = 40 ppm, [HOBT] = 50 μM, [H_2_O_2_] = 0.1 mM. Click here for file

Additional file 4: Figure S4 Effect of pH on SBP/H_2_O_2_/HOBT mediated degradation of CP6R. [CP6R] = 40 ppm, [HOBT] = 50 μM, [H_2_O_2_] = 0.1 mM, [SBP] = 0.27 μM. Click here for file

Additional file 5: Figure S5 Proposed mechanism for the oxidation of the diketo intermediate (A1) to dicarboxylic acid intermediate (A2). Click here for file
