# Supplementary figures and images for: Soybean peroxidase-mediated degradation of an azo dye– a detailed mechanistic study
Source: BMC Biochem. 2013 Dec 5;14:35. doi: 10.1186/1471-2091-14-35 (PMC4028748; doi:10.1186/1471-2091-14-35)

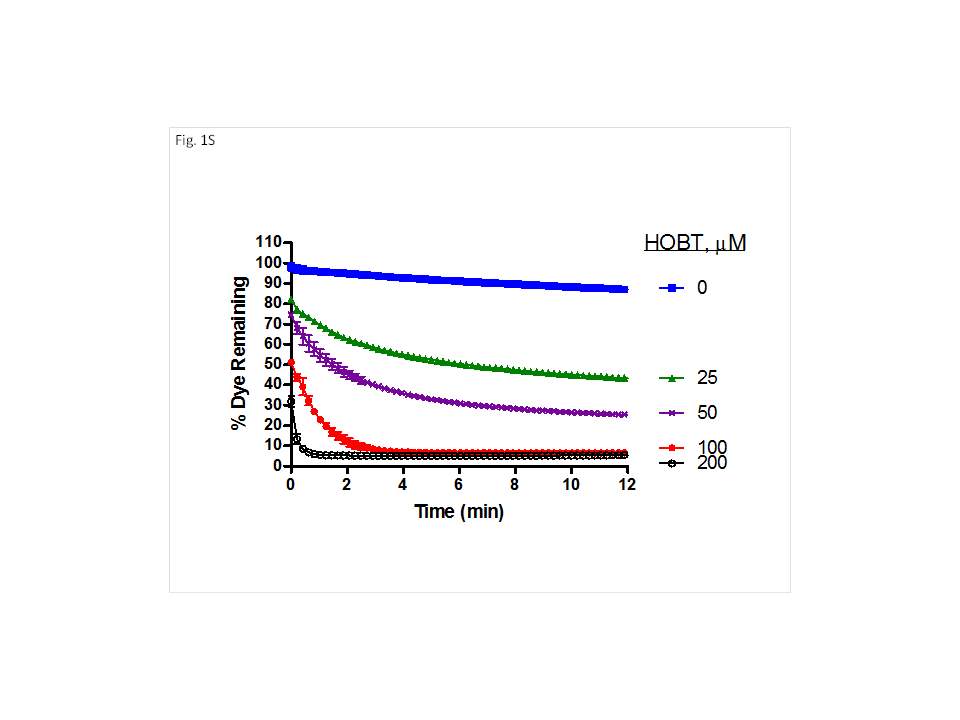

Supplement: Additional file 1: Figure SI — Effect of HOBT concentration of SBP/H2O2 mediated degradation of CP6R. [CP6R] = 40 ppm, [SBP] = 032 μM, [H2O2] = 0.1 mM. [file 1471-2091-14-35-S1.tiff]

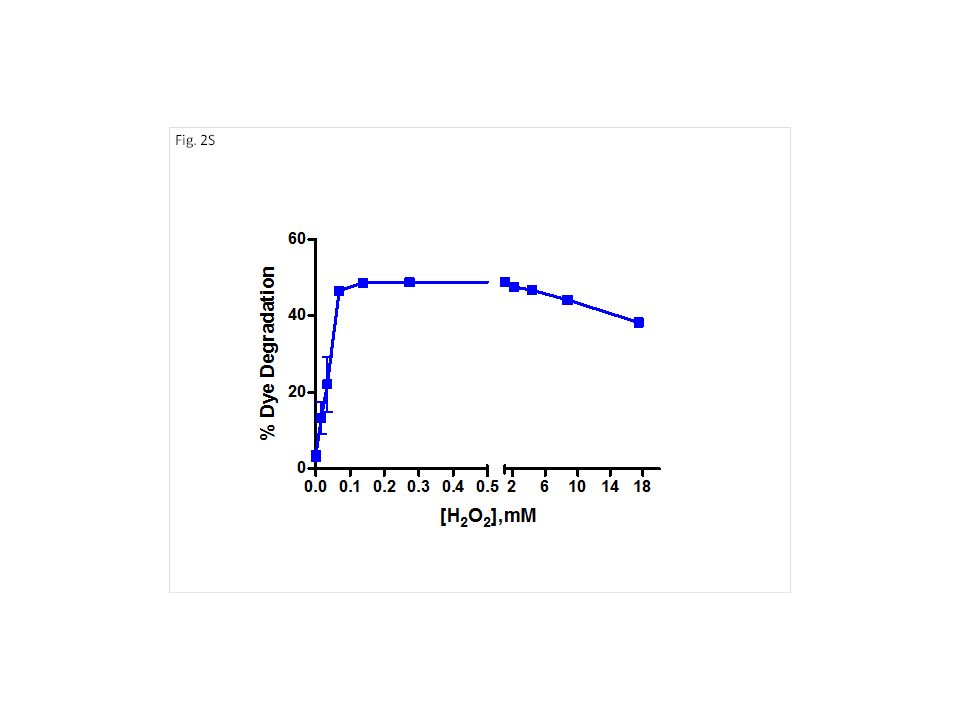

Supplement: Additional file 2: Figure S2 — Effect of H2O2 concentration on SBP/H2O2/HOBT mediated degradation of CP6R. [CP6R] = 40 ppm, [SBP] = 032 μM, [HOBT] = 50 μM. [file 1471-2091-14-35-S2.tiff]

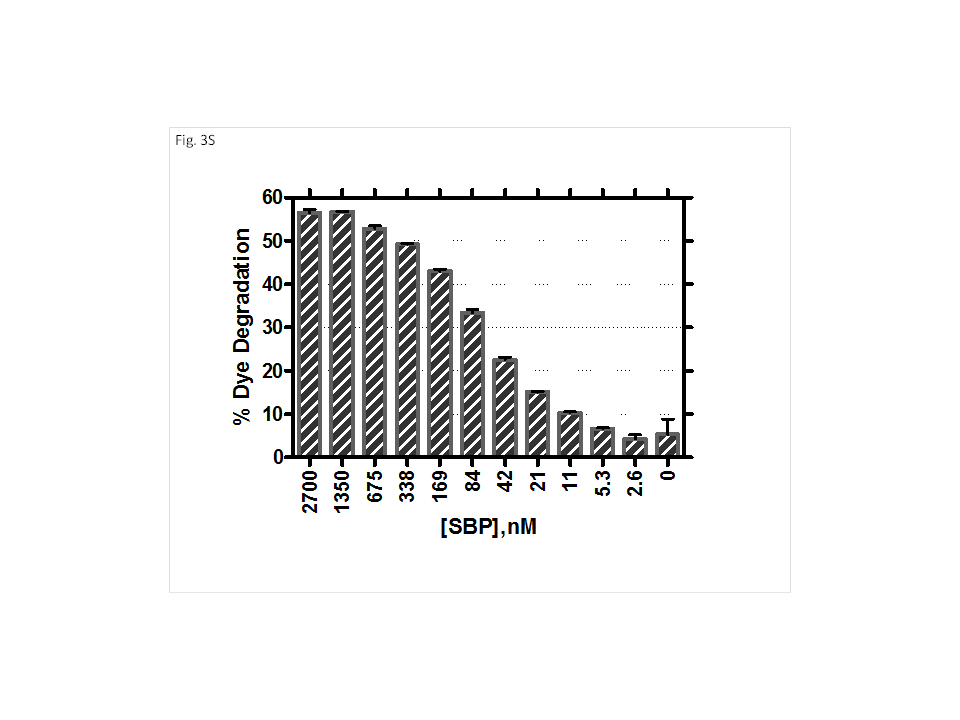

Supplement: Additional file 3: Figure S3 — Effect of SBP enzyme concentration on SBP/H2O2/HOBT mediated degradation of CP6R. [CP6R] = 40 ppm, [HOBT] = 50 μM, [H2O2] = 0.1 mM. [file 1471-2091-14-35-S3.tiff]

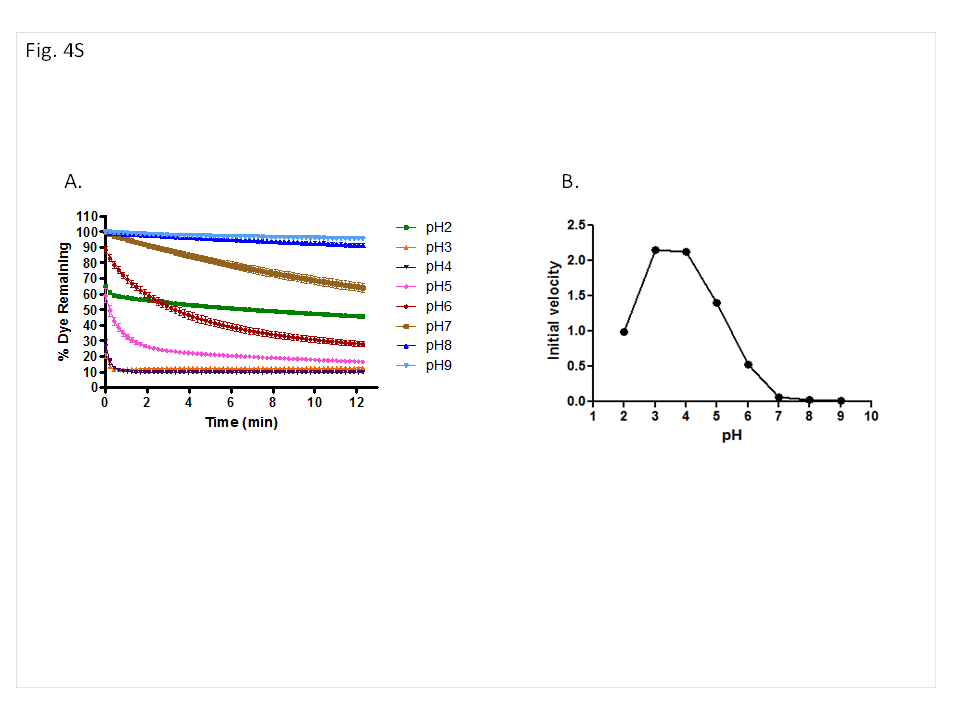

Supplement: Additional file 4: Figure S4 — Effect of pH on SBP/H2O2/HOBT mediated degradation of CP6R. [CP6R] = 40 ppm, [HOBT] = 50 μM, [H2O2] = 0.1 mM, [SBP] = 0.27 μM. [file 1471-2091-14-35-S4.tiff]
